# CAPABLE FAMILY FOR THE CAREGIVER–CARE RECIPIENT DYAD WITH DEMENTIA AND DISABILITY

**DOI:** 10.1093/geroni/igae098.0588

**Published:** 2024-12-31

**Authors:** Emerald Jenkins

**Affiliations:** Johns Hopkins University, Baltimore, Maryland, United States

## Abstract

Co-occurring physical and cognitive impairments in older adults with mild cognitive impairment (MCI)/early dementia lead to decreased functional independence and increased mortality. The purpose of this study is to address the needs of older adults with co-occurring physical disability and MCI/early dementia and their caregivers by adapting the Community Aging in Place, Advancing Better Living for Elders (CAPABLE) program to become CAPABLE-Family. To adapt CAPABLE, we performed a two-part multi-method study, part included: (1) semi-structured interviews with clinicians (n= 15) and (2) photovoice/semi-structured interviews (n= 12) of persons with mild cognitive impairment (MCI)/early dementia and their care partners (dyads). The second part of the study was an open label pilot to (1) adapt CAPABLE for persons with co-occurring physical disabilities and mild cognitive impairment (MCI)/early dementia (n= 7) and their care partners (n= 6) (dyads), and (2) evaluate acceptability/feasibility of the CAPABLE-Family intervention. The pilot included both qualitative and quantitative descriptive analysis. Findings from clinician interviews include, for example: incorporate a greater emphasis on safety, memory, nutrition and advanced care planning. Findings from the photovoice were categorized into 3 major themes. From the pilot, difficulty with ADLs and IADLs improved in older adults. Depression and pain interference improved in care partners. Qualitative feedback from the dyads in the open label pilot included, for example: the importance of written/tangible items. There are multiple methods for mood mitigation, self-esteem, and memory. Evidence from this research points to the importance of individualized intervention/care elements for this population.

